# Clinical and economic evaluation of risk factor guided respiratory syncytial virus prophylaxis in Colombian preterm infants

**DOI:** 10.1186/s12962-025-00710-z

**Published:** 2026-01-27

**Authors:** Carlos E. Rodriguez-Martinez, Jaime Ordonez, Xavier Carbonell-Estrany, John Fullarton, Ian Keary, Barry Rodgers-Gray, Ivonne D’Apremont, Daniel E. Noyola, Paulo Andre Ribeiro, Renato T. Stein, Nestor Vain, Jean-Eric Tarride, Bosco Paes

**Affiliations:** 1 Department of Pediatrics, Universidad Nacional de Colombia, Bogota, Colombia; 2 True Consulting, Medellín, Colombia; 3 Neonatology Service, Hospital Clinic, Barcelona, Spain; 4 Violicom Medical Limited, Aldermaston, United Kingdom; 5 Department of Neonatology, Pontificia Universidad Católica de Chile, Santiago de Chile, Chile; 6 Microbiology Department, Universidad Autónoma de San Luis Potosí, San Luis Potosí, México; 7 Department of Neonatology, Centro Hospitalar Unimed - Unimed Joinville, Joinville, Brazil; 8 Social Responsibility, Hospital Moinhos de Vento and Department of Pediatrics, Pontifícia Universidade Católica do Rio Grande do Sul, Porto Alegre, Brazil; 9 Neonatolgy, Hospital Sanatorio Trinidad Palermo, Buenos Aires, Argentina; 10 Centre for Health Economics and Policy Analysis and Department of Health Research Methods, Evidence and Impact, Mcmaster University, Hamilton, Ontario Canada; 11 Programs for Assessment of Technology in Health, St Joseph’s Healthcare, Hamilton, Canada; 12 Department of Pediatrics, Mcmaster University, Hamilton, Ontario Canada

**Keywords:** International risk scoring tool, RSV, Palivizumab, Prophylaxis, Cost-effectiveness

## Abstract

**Background:**

The International Risk Scoring Tool (IRST) comprises three risk factors (age relative to the respiratory syncytial virus [RSV] season; household and/or maternal smoking; siblings and/or daycare attendance) and enables the cost-effective targeting of palivizumab to infants born at 32–35 weeks’ gestational age (wGA) at greatest risk of related hospitalization (RSVH). This study provides the first evaluation of IRST-guided RSV prophylaxis in Colombia.

**Methods:**

The IRST (with wGA substituted for chronological age as RSV is endemic in Colombia) plus other risk factors were assessed using data from 81 infants born 32–35 wGA with RSVH and 49 gestational- and age-matched controls. A cost-utility model comparing IRST-guided palivizumab versus no prophylaxis from the healthcare provider perspective was then adapted using Colombian costs and data (5% discounting). Infants could experience either RSVH, emergency room medically-attended, RSV infection, or remain uninfected/non-attended, with possible long-term respiratory morbidity up to 18 years of age over a lifetime time horizon.

**Results:**

The most predictive combination of risk factors was the IRST plus mixed breast and formula feeding and maternal education (area under the receiver operating characteristic curve = 0.823). For infants assessed at moderate- and high-risk of RSVH, the cost/quality-adjusted life year (QALY) was COP20,225,126 (USD4,752). Probabilistic sensitivity analyses resulted in a mean of COP22,193,734/QALY (USD5,214/QALY), with a 61.1% probability of palivizumab being cost-effective at a COP28,193,734 (USD6,624; 2022 gross domestic product/capita) willingness-to-pay threshold.

**Conclusions:**

Palivizumab prophylaxis of 32–35 wGA infants at moderate- and high-risk of RSVH, identified using the Colombian-adapted IRST, proved to be cost-effective versus no intervention.

**Supplementary Information:**

The online version contains supplementary material available at 10.1186/s12962-025-00710-z.

## Introduction

Respiratory syncytial virus (RSV) is a major global disease of early childhood causing approximately 12.9 million lower respiratory tract infections (LRTIs), 2.2 million related hospitalizations (RSVHs), and 66,500 deaths each year in infants [1]. Importantly, more than 95% of RSV-LRTI episodes and 97% of RSV-related deaths occur in low- and middle-income countries (LMICs) [1]. In Colombia, studies of infants hospitalized with LRTI have reported that between 26 and 38% were RSV-positive [2, 3], with associated mortality observed only in those with confirmed RSV infection [2]. The well-established high-risk populations with chronic lung disease (CLD), congenital heart disease (CHD), and prematurity who are at risk for RSVH, were identified in these Colombian cohorts [2–6]. At present, there is no effective treatment for RSV-LRTI so management is aimed at prevention either through hygiene measures or immunization.

Immunoprophylaxis with palivizumab, a humanized monoclonal antibody, has proven highly effective at preventing RSVH in high-risk infants, such as those with CLD, CHD, and premature birth at ≤ 35 weeks’ gestational age (wGA) [7–9]. A prospective, observational study of 600 Colombian infants born at ≤ 32 wGA or with CLD or CHD reported that palivizumab was well-tolerated and reduced RSVHs by an estimated 86–93% and RSV-related mortality by 97% [10]. Palivizumab is currently the only preventive intervention available in Colombia, however, its use is restricted to < 32 wGA infants and those born 32–34 wGA with CLD, primarily due to cost considerations [11, 12]. An economic analysis from 2013, commissioned by the Ministry of Health and the National Guideline for Integral Attention of Preterm Infants Study Group, determined palivizumab to be not cost-effective *versus* no prophylaxis in infants < 35 wGA (incremental cost-effectiveness ratio [ICER] *per* quality adjusted life year [QALY] gained of COP53,121,137 [USD12,481]) [11]. Conversely, a recent analysis found palivizumab to be a dominant strategy over no prophylaxis for preventing RSVH in preterm neonates ≤35 wGA and infants with CLD and CHD [13]. To date, no analysis has assessed the cost-effectiveness of palivizumab specifically in infants born 32–35 wGA.

For otherwise healthy infants born 32–35 wGA, targeting palivizumab prophylaxis to those at greatest risk of RSVH using the International Risk Scoring Tool (IRST) has proven a cost-effective strategy in both Canada and Italy [14–16]. The IRST comprises three risk factors (birth 3 months before to 2 months after the RSV season start date; household and/or maternal smoking; siblings and/or daycare attendance) to accurately categorize the risk of RSVH in these infants (Figure S1 & Table S1) [14]. The IRST was developed and validated using data from seven studies of 14,553 infants born 32–35 wGA from high-income, Northern Hemisphere countries with a defined RSV season [14]. For adoption in Colombia, the IRST requires modification to reflect the tropical climate where RSV has an endemic, non-seasonal pattern, with increased epidemic activity associated with rainy periods [17], and where the importance (weighting) of the other risk factors may differ within the context of an upper middle-income country. The objective of this study was to generate a Colombian-specific version of the IRST and to assess whether its adoption in Colombia could guide the cost-effective use of palivizumab (*versus* no prophylaxis) in 32–35 wGA infants.

## Materials and methods

### Development of a Colombian-specific version of the IRST

Risk factor data were collected from otherwise healthy infants born at 32 weeks 0 days to 35 weeks and 6 days between November 2022 and February 2023 at two hospitals in Colombia (Fundación Hospital Pediátrico la Misericordia (HOMI) and Clínica de Marly Jorge Cavelier Gaviria). Infants had either an RSVH within the first six months of life (cases) or were gestationally and chronologically age-matched controls born at the participating hospitals but with no RSVH or other respiratory illness related hospitalization. Infants with significant morbidity other than prematurity, such as CLD, CHD and immunodeficiency, neuromuscular impairment, Down syndrome and cystic fibrosis were excluded. Information on the IRST risk factors: smokers in the household (Yes/No), maternal smoking (Yes/No), siblings (Yes/No) and daycare attendance (Yes/No) were collected. In addition, as a potential substitute for ‘birth 3 months before to 2 months after the RSV season start date’ within the original IRST, data on wGA (32, 33, 34, 35 wGA) together with two other risk factors considered important in Latin America (LATAM), breastfeeding (defined as exclusive and given or planned from birth to 3 months of age, or mixed [breastfeeding and formula]; Yes/No), and maternal education to primary level or less (as a surrogate for social deprivation; Yes/No), were investigated. Analysis of the pooled dataset underpinning the IRST revealed that, whilst not reaching statistical significance, an expected trend towards increased rates of RSVH with lower wGA at birth (Table S2) was present; hence, arose the rationale for exploring whether wGA had the discriminatory power to be a substitute for chronological age in relation to the RSV season start date in a Colombian version of the IRST. Predicated on previous RSV risk factor model assessments [18–20], a total of 100 infants (50 cases and 50 controls) with complete risk factor data and who had not received palivizumab (or any other RSV preventive intervention) were targeted. The study protocol was approved by the ethics committee of the Fundación Hospital de La Misericordia.

The Colombian risk factor data were initially subjected to logistic regression, with all variables treated as categoric, to generate the equivalent of the IRST, with wGA substituted for chronological age, and subsequently with and without the addition of breastfeeding (exclusive or mixed) and maternal education either singly or combined with the other risk factors. For each analysis, predictive accuracy was assessed by calculation of the area under the receiver operating characteristic curve (AUROC), with a value of ≥0.75 considered ‘good’ (original IRST AUROC: 0.773) [14]. The combination of Colombian risk factors with the highest AUROC was then selected for further analysis.

In the original IRST, infants classified as low-risk (score of ≤19) had a 1.0% RSVH rate, moderate-risk (score of 20–45) a 3.3% rate, and high-risk (score of 50–56) a 9.5% rate (Table S3) [14]. The combined moderate- and high-risk groups had a 6.3% RSVH rate and captured 85.2% of all RSVHs [14]. To create the final Colombian-specific version of the IRST, the RSVH rates for each risk group were adjusted using a *pro rata* interpolation from the IRST to the Colombian data, predicated on the predictive accuracy of the selected Colombian model. This assumed the same number of total RSVHs across the risk groups for the IRST and Colombian-specific IRST and, in the absence of any published Colombian epidemiological data for 32–35 wGA infants, an estimated RSVH rate of 7.6%. The latter was based on a single arm study of palivizumab prophylaxis in < 35 wGA infants (40.1% with CLD) that reported an RSVH rate of 1.9% (no RSVHs in 33–34 wGA infants) uplifted to reflect an assumed effectiveness of 75% risk reduction [10, 21, 22]. RSVH rates are presented as median with 95% confidence intervals (CI).

### Cost-effectiveness of the Colombian-specific version of the IRST at guiding palivizumab prophylaxis

#### Cost-utility model overview

**Fig. 1:**
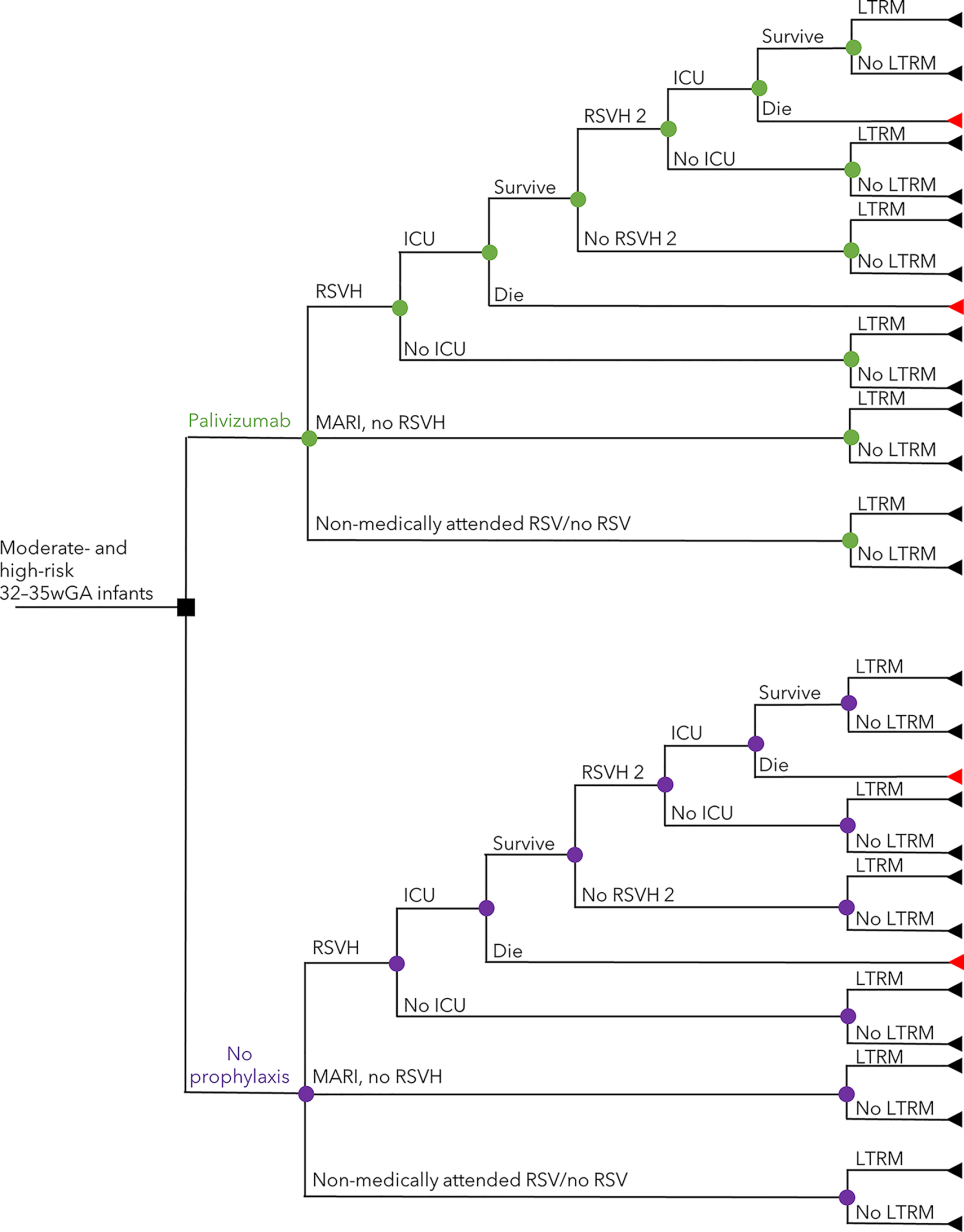
Decision tree describing the clinical pathway utilized within the model. Nodes represent points where more than one event is possible; the square node represents a decision addressed by the model. Branches represent possible events experienced by patients. Triangles represent decision tree endpoints. ICU intensive care unit, LTRM long-term respiratory morbidity, MARI medically-attended RSV infection without hospitalization, RSV respiratory syncytial virus, RSVH RSV-related hospitalization.

A previously published and validated Canadian cost-utility model, that assessed IRST-guided palivizumab prophylaxis *versus* no prophylaxis [15], was adapted to the Colombian healthcare provider perspective. In brief, the model included a decision-tree wherein prophylaxed/non-prophylaxed infants at moderate- or high-risk of RSVH, as scored by the Colombian IRST, could experience either RSVH, emergency room medically-attended RSV infection (MARI) [23], or remained uninfected/non-attended (Fig. 1). Palivizumab efficacy (82.2% relative reduction in RSVH) for 32–35wGA infants was drawn from the IMpact-RSV trial (Table 1) [22]. The RSVH rate for infants assessed at moderate- or high-risk not receiving prophylaxis was as *per* the final Colombian-specific IRST. Infants with RSVH (mean stay 8.6 days) could be admitted to the intensive care unit (ICU; 7.8%) and suffer potential mortality (3.6%), commensurate with published Colombian data [2, 24, 29]. All surviving infants, irrespective of RSV infection status, had the potential to experience respiratory morbidity for up to 18 years of age over a lifetime horizon (Table S4). In the absence of any Colombian-specific data or such data from a country similar in terms of healthcare systems, genetic backgrounds and socioeconomic conditions, long-term morbidity was modelled using Spanish [30] and Swedish data [31–33]. The impact of palivizumab was then applied using data from three studies: Simoes et al. [34], Blanken et al. [35] and Yoshihara et al. 2013 [36]. Utility values for RSVH, MARI and for respiratory morbidity were unchanged from the Canadian cost-utility analysis, with the rates for each respective variable for the prophylaxed and non-prophylaxed infants used as multipliers to calculate aggregated utilities for the two populations (Table 1) [15, 25–28].

**Table 1:** Input parameters for the cost-utility model

Parameter	Point estimates		Reference source(s)
	Palivizumab	No palivizumab	
**Palivizumab efficacy (RRR)**	82.2%	-	Notario et al. 2014 [22]
**RSVH**^**a**^			
- Overall rate	3.3%	18.6%^f^	Colombian-specific IRST
- Ward LOS, mean days	8.6	8.6	Piñeros et al. 2013 [2]
- ICU rate	7.8%	7.8%	Rodriguez-Martinez et al. 2020 [24]
- Utility in hospital	0.60	0.60	Weiner et al. 2012 [25] and Leidy et al. 2005 [26]
- Utility post discharge			
- No sequelae	0.88	0.88	Greenough et al. 2004 [27]
- Long-term sequelae^b^	0.79	0.79	Chiou et al. 2005 [28]
- Mortality^c^	3.6%	3.6%	Villamil et al. 2020 [29]
**MARI**			
- Rate^d^	2.95%	16.57%	Carbonell-Estrany et al. 2010 [23]
- Utility no sequelae or respiratory symptoms	0.95	0.95	Greenough et al. 2004 [27]
Utility long-term sequelae or respiratory symptoms^e^	0.79	0.79	Chiou et al. 2005 [28]
**No RSV**^**e**^			
Utility No sequelae or respiratory symptoms	0.95	0.95	Greenough et al. 2004 [27]
Utility long-term sequelae or respiratory symptoms^e^	0.79	0.79	Chiou et al. 2005 [28]

^a^First and subsequent RSVHs. ^b^ Long-term respiratory morbidity following RSVH or MARI was assumed to last until age 18, but only until age 6 in infants without an RSV infection or who had a non-medically attended RSV infection, using the same rates as for MARI (Table S4). ^c^ Applied only to patients in ICU. ^d^ Emergency department only. ^e^ Infants without an RSV infection or not requiring medical management. ^f^S ee Table 3 for full details. ICU intensive care unit, LOS length of stay, MARI medically-attended RSV infection without hospitalization, RRR relative risk reduction, RSV respiratory syncytial virus, RSVH RSV-related hospitalization, wGA weeks’ gestational age

#### Costs

Pecuniary inputs included those for RSVH (including ICU admission), MARI, ongoing respiratory morbidity, and societal costs, in addition to both acquisition and administration costs relating to palivizumab in Colombia (Table 2). The total average cost for palivizumab *per* 32–35 wGA infant was COP7,790,674 (USD1,830). This assumed an average of 4 injections for all infants irrespective of date of parturition since RSV is endemic (50 mg vial: COP1,416,724 [USD333]; 100 mg vial: COP2,804,887 [USD659] [37]) with 70% vial sharing (5% wastage) [Note: palivizumab vials are single use only], in line with local clinical practice (Dr Rodriguez-Martinez) and studies [10, 21], and a weight at each administration calculated by applying a growth algorithm [41] to an estimated average birth weight of 1991 g [42, 43]. Administration costs assumed 70% of injections were given by a nurse and 30% by a clinician, resulting in a blended cost of COP72,812 [USD17] *per* infant [24, 38]. The annual cost of long-term respiratory morbidity *per* child was COP2,229,920 (USD524), representing a cost of COP3,428,872 (USD806) for 0–6 years of age [39] and 1,630,444 (USD383) for 7–18 years of age [40], using Colombian age-group specific wheezing and asthma data.

**Table 2:** Direct costs for cost-utility model

Parameter	Cost (COP)	Units	Reference source(s)
**Palivizumab**			
- 50 mg vial - 100 mg vial	1,416,724.00 2,804,887.19	Lowest combination of vials *per* infant weight	Calculated from prices paid data [37]
- Administration - Clinician - Nurse specialist	32,450.23 12097.00	1 *per* injection, assuming 30% clinician and 70% nurse administered	Rodriguez-Martinez et al. 2020 [24] Economic Research Institute [38]
**RSVH total stay (including preadmission and ICU costs)**	7,240,740.14^c^	1 *per* RSVH^b^	Rodriguez-Martinez et al. 2020 [24]
**MARI** - ED visit	69,663.41	1 *per* affected infant^a^	Rodriguez-Martinez et al. 2020 [24]
**Respiratory morbidity (per annum)**	2,229,919.86	1 *per* affected infant^b^	Years 0–6: Buendía et al. 2022 [39] Years 7–18: Flóres-Tanus et al. 2018 [40]

Costs uplifted as required to Colombia 2022 levels using the latest World Bank Data available at the time of writing. ^a^See Table 1 for rates. ^b^See Table S4 for rates. ^c^Specific cost for preterm infants without CLD

All costs were derived from publicly available sources and, if required, adjusted for inflation to Colombia 2022 levels using the latest World Bank Data available at the time of writing. A 5.0% discount was applied to costs and utilities as *per* Colombian recommendations [44].

#### Outputs

Outcomes were expressed as the cost *per* QALY (incremental cost-utility ratio [ICUR]) for palivizumab *versus* no intervention [44]. The willingness-to-pay (WTP) threshold in Colombia was determined to be COP28,193,734 (USD6,624), the gross domestic product (GDP) *per capita* [45].

#### Sensitivity analyses

Probabilistic (PSA) and deterministic (DSA) sensitivity analyses were performed with limits of plus or minus 10% and 20%, respectively, applied on the values of the tested variables. The PSA (10,000 Monte Carlo simulations) used gamma distribution as the default for costs, beta distribution for utilities and RSVH and associated rates, and normal distribution for discount rates and mortality rate (as an approximation of Poisson distribution) (Table S5).

#### Scenario analyses

The following scenario analyses were performed: i) inclusion of indirect/societal costs, which were adapted from a Colombian study on pediatric cutaneous leishmaniasis [46] in the absence of any RSV-specific data (Table S6); ii) no vial sharing; iii) attenuating the duration of long-term respiratory morbidity at 6 or 13 years of age.

#### Software

All analyses and modelling were undertaken using WinBUGS, SPSS and Microsoft Excel 365.

## Results

### Colombian-specific version of the IRST

**Fig. 2:**
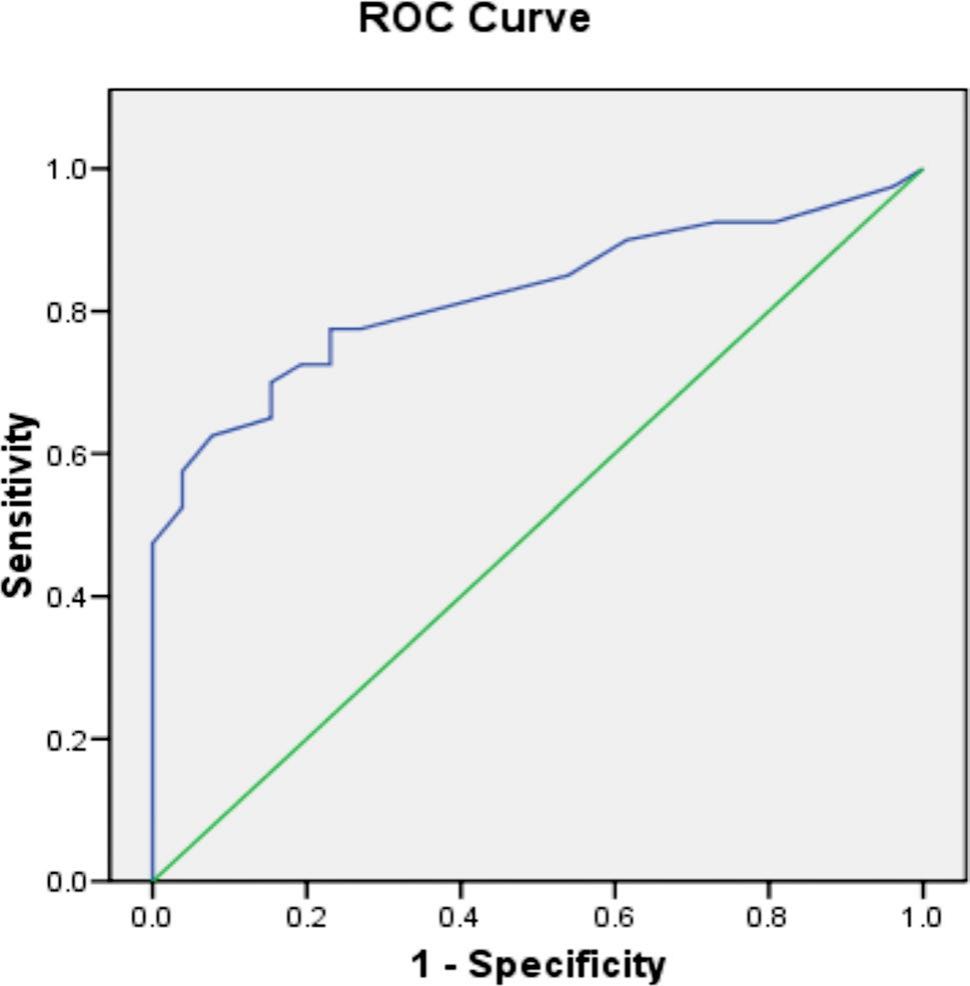
Predictive accuracy of the Colombian-specific version of the IRST. AUROC: 0.823 (95%CI 0.723 – 0.923). Included risk factors: Gestational age; smokers and/or maternal smoking; siblings and/or daycare attendance; mixed breastfeeding (lack of breastfeeding plus formula); maternal education to primary level or less. AUROC Area under the receiver operating characteristic curve, with 1 representing perfect predictive accuracy, IRST International Risk Scoring Tool

Data on 81 cases and 49 controls were collected (Table S7). Generating the IRST using Colombian data with wGA substituting for chronological age resulted in an AUROC of 0.751 (Figure S2). Predictive accuracy was improved by adding lack of exclusive breastfeeding or mixed breastfeeding, with the latter providing the higher AUROC (0.791 vs 0.803, respectively; Figure S3 & Figure S4). Adding maternal education to primary level together with mixed breastfeeding proved the most predictive combination with an AUROC of 0.823 (95% CI 0.723–0.923; Fig. 2). Within this analysis, wGA contributed approximately 16% to the overall discrimination between infants with *versus* without RSVH.

The RSVH rate for the moderate- and high-risk group in the Colombian-specific IRST was recalculated to be 18.6% (Table 3).

**Table 3:** RSVH rates for each risk group in the Colombian-specific IRST

Risk group	RSVH risk, median % (95% CI)
High	41.0% (22.7–100.0%)
High and moderate	18.6% (12.3–31.9%)
Moderate	7.4% (3.6%–16.4%)
Low	1.8% (0.7–3.0%)

*CI* Confidence interval, *IRST* International Risk Scoring Tool, *RSVH* respiratory syncytial virus hospitalization

### Cost-effectiveness of the Colombian-specific version of the IRST at guiding palivizumab prophylaxis

For 32–35wGA infants scored at moderate- and high-risk by the Colombian-specific IRST, palivizumab increased treatment costs, but resulted in reduced direct and indirect healthcare costs and improved quality of life compared to no prophylaxis (Table 4). The resultant ICUR/QALY was COP20,225,126 (USD4,752), over 25% below the Colombian WTP threshold of COP28,193,734 (USD6,624).

**Table 4:** Incremental costs and utilities for palivizumab *versus* no prophylaxis using the Colombian-specific IRST to identify 32–35wGA infants at moderate- and high-risk of RSVH

	**No Palivizumab**	**Palivizumab**	
Treatment costs (COP)	0.00	7,790,674.32	
Direct costs (COP)	4,576,068.98	1,077,500.22	
Difference in costs (COP)	4,292,105.56		
QALYs	20.207		20.419
Difference in QALYs	0.212		
**ICUR (COP) per QALY gained**	**20,225,126.34**		

*COP* Colombian peso*, ICUR* incremental cost utility ratio*, IRST* international risk scoring tool*, QALY* quality adjusted life year*, RSVH* respiratory syncytial virus hospitalization, *wGA* weeks’ gestational age

### Sensitivity analyses

**Fig. 3:**
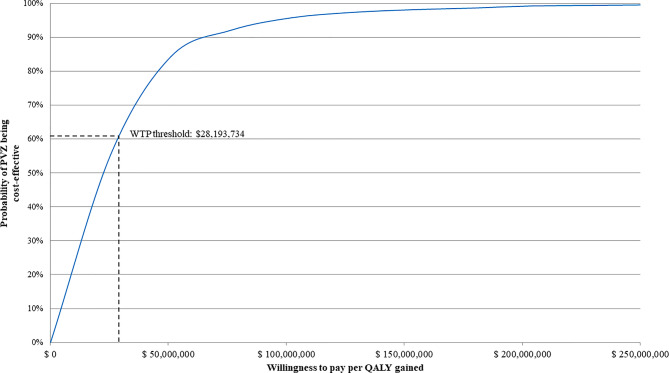
Cost-effectiveness acceptability curve for palivizumab prophylaxis (vs no prophylaxis) in moderate- and high-risk 32–35 wGA infants identified by the Colombian-specific IRST. Results are based on the probabilistic analysis after 10,000 Monte Carlo simulations. The curve represents the difference in costs and benefits between prophylaxis and no prophylaxis. COP Colombian peso, IRST international risk scoring tool, PVZ palivizumab, QALY quality-adjusted life year, wGA weeks’ gestational age

The PSA resulted in a mean ICUR of COP22,193,734/QALY (USD5,214), with an 61.1% probability of palivizumab being cost-effective at a COP28,193,734 (USD6,624) WTP threshold (Fig. 3 and Figure S5). In the DSA, the cost-utility model was found to be most sensitive to: the mean number of palivizumab injections; palivizumab cost (100 mg); utility scores; RSVH rate; and palivizumab efficacy (Figure S6).

### Scenario analyses

Inclusion of societal costs improved the ICUR/QALY gained for palivizumab *versus* no prophylaxis to COP18,282,582 (USD4,296) (Table 5). Conversely, removing vial sharing and attenuating the duration of long-term respiratory morbidity to 6 or 13 years of age increased the ICUR/QALY to COP29,059,655 (USD6,828), COP38,015,084 (USD8,932), and COP26,350,141 (USD6,191), respectively, albeit the latter was still under the Colombian WTP threshold (COP28,193,734 [USD6,624]).

**Table 5:** Results of scenario analyses

	ICUR per QALY gained (COP)	
Scenario	As per scenario	Base case
Inclusion indirect/societal costs *Base case: direct costs only*	18,282,582.13	20,225,126.34
No vial sharing *Base case: 70% vial sharing (5% wastage)*	29,059,654.66	
13 years duration of long-term respiratory morbidity in affected infants 6 years duration of long-term respiratory morbidity in affected infants *Base case: 18-year respiratory morbidity*	26,350,141.17 38,015,084.47	

*COP* Colombian peso, *ICUR* incremental cost-utility ratio, *QALY* quality adjusted life year, *RSVH* respiratory syncytial virus hospitalization

## Discussion

In this first assessment of the IRST in LATAM, a localized version of the IRST was found to be highly predictive at determining the risk of RSVH in Colombian 32–35 wGA infants. Utilization of this Colombian-specific version of the IRST to target palivizumab prophylaxis to 32–35 wGA infants at moderate- or high-risk of RSVH was subsequently demonstrated to be a cost-effective strategy (*versus* no prophylaxis) from the perspective of the Colombian healthcare system. These results support the relevance of the IRST across LATAM and that use of the Colombian version should be adopted and assessed prospectively to guide palivizumab prophylaxis to 32–35 wGA infants in routine clinical practice in Colombia.

Generating a Colombian version of the IRST, with wGA substituted for chronological age, resulted in an AUROC of 0.751, which compared very favourably with an AUROC of 0.773 for the original IRST and 0.707 in the validation exercise using data from the RSV Preterm Risk Estimation Measure for RSVH in Ireland study (PREMI) [14, 47]. Importantly, our results indicate that in endemic (non-seasonal) RSV environments, such as in Colombia, wGA may be a suitable substitute for birth relative to the RSV season start. The Colombian version of the IRST was further refined by adding maternal education to primary level (as a surrogate for social deprivation) together with mixed breastfeeding, two risk factors considered of particular relevance in LATAM, producing a highly predictive AUROC of 0.823. Social deprivation is known to be associated with poorer health outcomes and more limited access to healthcare, particularly in LMICs [48], as well as being a specific risk factor for RSVH [49]. Similarly, lack of breastfeeding is well-recognized as an important risk factor for RSVH in 32–35 wGA infants and, indeed, was the next most predictive variable apart from those included in the original IRST [50, 51]. In addition to increasing the predictive power, the inclusion of mixed breastfeeding in the Colombian IRST provides an opportunity to encourage exclusive breastfeeding for these premature infants, in line with WHO recommendations [52]. The RSVH rate for the moderate- and high-risk group in the Colombian-specific IRST was 18.6%, which is similar to that reported for infants with CLD [4], who receive routine palivizumab prophylaxis in Colombia [12].

We recognize that the risk factor data collected in this study is limited both in terms of including only two Colombian hospitals over a brief, 4-month period and a relatively small sample size (81 cases; 49 controls). This may have resulted in overperformance of the Colombian-specific IRST (AUROC 0.823) and wide confidence intervals (CIs) on the RSVH rates, particularly for the high-risk group (95% CI 22.7–100.0%). Albeit the latter is somewhat mitigated by the fact that the cost-utility analyses were predicated on infants classified as moderate- or high-risk for RSVH where the uncertainty was more constrained (95% CI 12.3–31.9%). The substitution of chronological age for gestational age engenders a different predictive mechanism, from seasonal timing (including immune maturation, declining maternal antibodies, and exposure patterns) to static developmental status at birth, respectively, and requires further study and confirmation of validity. Despite these caveats, this study has demonstrated the value of undertaking localization of the IRST for populations that are not well represented in the underlying dataset. The improved predictive power engendered by localizing the IRST enables prophylaxis to be better targeted to those 32–35 wGA infants at greatest risk of RSVH specifically in Colombia. It is recommended that a larger prospective validation be included as part of the deployment of the IRST in any other country.

When the Colombian-specific IRST was used to identify 32–35 wGA infants at moderate- and high-risk of RSVH for prophylaxis, a new cost-utility analysis produced an ICUR/QALY of COP20,225,126 (USD4,752) (mean: COP22,193,734/QALY [USD5,214]) for palivizumab *versus* no prophylaxis. This equated to a 61.1% probability of palivizumab being cost-effective against a WTP threshold of COP28,193,734 ([USD6,624], the Colombian GDP *per capita*). Of note, palivizumab was borderline cost-effective with no vial sharing (COP29,059,655 [USD6,828]) and was below the WTP threshold in an analysis where respiratory morbidity was curtailed at 13 years (COP26,350,141 [USD6,191]). It is now widely acknowledged that long-term respiratory morbidity following RSVH in infancy can extend throughout childhood [33, 53]; hence, this scenario can be viewed as conservative.

It is difficult to compare our cost-utility analysis to those previously published for palivizumab use in Colombia, one of which found prophylaxis not to be cost-effective [11] and the other dominant (cost saving) [13], due to several salient differences in the comparative model structures, assumptions and inputs. Most importantly, our analysis focused on healthy 32–35 wGA infants at moderate- or high-risk of RSVH as identified by the Colombian-specific IRST, whereas the previous models assessed all < 35 wGA infants (with [11] or without CLD [13]). This resulted in differing RSVH rates being used in the analyses, further amplified by the limited epidemiological data on RSV in Colombia. In the analysis from 2013 [11], the RSVH rate of 10.6% was drawn from the IMpact study [7], whereas the 2024 study used a very high rate of 34.9% derived from hospitalized infants in the SENTINEL1 study [13, 54]. Due to the aforementioned lack of Colombian epidemiological data, we assumed an RSVH rate of 7.6% in our analysis, which was used to adjust the RSVH risk for the low-, moderate- and high-risk groups within the IRST. A 7.6% RSVH rate can be considered reasonable and somewhat conservative with reports of rates between 10 and 12% in preterm infants < 36–37 wGA from Brazil and Peru, albeit these studies included those with CLD who were born < 32 wGA [55, 56]. Further studies are required to quantify the true burden of RSV in healthy Colombian 32–35 wGA infants. Other differences between our analysis and the two previous publications include the efficacy rates used for palivizumab drawn from the IMpact study [7, 21] (82.2% in our model for 32–35 wGA infants; 78.1% in the 2024 analysis for ≤35 wGA infants without CLD [13]; 54.7% in the 2013 analysis for ≤35 wGA infants including those with CLD) [11], and the inclusion (our analysis and 2024 analysis [13]) or not (2013 analysis [11]) of MARI, further compromise direct comparisons. Limitations for all three analyses include a universal lack of country-specific utility scores and long-term respiratory morbidity data [11, 13], which have been addressed in detail elsewhere [15]. In brief, whilst recent data have further elucidated the impact of RSV on QoL [57, 58] these data are derived from a much broader population of children than considered in our model and did not report a disutility for hospitalized infants. As such, the data from Greenough et al. [27] used herein arguably remain the best source of utilities following RSVH in premature infants. Similarly, utility data on the ongoing effect of long-term respiratory morbidity on a child’s health following RSVH is not available, necessitating the use of a surrogate value derived from a study of the QoL impact of asthma. Finally, whilst there is a growing body of evidence that palivizumab [34–36], and the newer long-acting monoclonals [59], can reduce long-term respiratory morbidity, there remains some degree of uncertainty about the size and, particularly, duration of effect. Utilization of data from three studies of palivizumab in our model minimizes some of this uncertainty [34–36].

Despite these limitations, confidence in the results of our cost-utility analysis comes from the ICUR/QALY (COP20,225,126 [USD4,752]) not only being lower than the Colombian cost-effectiveness threshold of 1 GDP *per capita* derived from the World Bank [45] (COP28,193,734 ([USD6,624]) but also that reported by the Central Bank of Colombia [60] (COP26,222,387 [USD6,161]), and two alternative WTP thresholds that have been postulated (USD5,043 [61] and USD6,060 [62]). Under the guidance from the World Health Organization (WHO) [63], our base case can be considered ‘highly cost-effective’, being under 1 GDP, with the more conservative scenarios (including when respiratory morbidity was curtailed at 6 years of age) classed as ‘cost-effective’, falling withing the range 1–2x GDP. These facts, combined with the sensitivity and scenario analyses conducted, enhance the validity of our findings against any uncertainty in the assumptions and inputs.

## Conclusion

Our study has confirmed that the Colombian specific IRST provides a potentially effective method to identify 32–35 wGA infants who are most likely to benefit from palivizumab prophylaxis. Furthermore, prophylaxis of these moderate- and high-risk 32–35 wGA infants with palivizumab was demonstrated to be cost-effective *versus* no intervention. These results also encourage the exploration and use of the IRST to guide the cost-effective use of palivizumab in 32–35 wGA infants across LATAM.

## Electronic supplementary material

Below is the link to the electronic supplementary material.

Supplementary material 1

## Data Availability

The datasets used and/or analyzed during the current study are available from the corresponding author on reasonable request.

## Abbreviations

AUROC: Area under the receiver operating characteristic curve
CHD: Congenital Heart Disease
CI: Confidence interval
CLD: Chronic Lung Disease
GDP: Gross domestic product
ICER: Incremental cost-effectiveness ratio
IRST: International Risk Scoring Tool
LATAM: Latin America, LMIC: Low- and middle-income countries
LRTI: Lower respiratory tract infection
QALY: Quality-adjusted life year
QoL: Quality of life
RSV: Respiratory syncytial virus
RSVH: Respiratory Syncytial Virus Hospitalization
wGA: Weeks’ gestational age
WTP: Willingness-to-pay

## References

[1] Li Y, Wang X, Blau DM, Caballero MT, et al. Global, regional, and national disease burden estimates of acute lower respiratory infections due to respiratory syncytial virus in children younger than 5 years in 2019: a systematic analysis. Lancet. 2022;399:2047–64. 35598608 10.1016/S0140-6736(22)00478-0 PMC7613574

[2] Piñeros JG, Baquero H, Bastidas J, et al. Respiratory syncytial virus infection as a cause of hospitalization in population under 1 year in Colombia. J Pediatr (rio J). 2013;89:544–48. 24029550 10.1016/j.jped.2013.04.002

[3] Rodríguez-Martínez CE, Sossa-Briceño MP, Nino G. Predictors of prolonged length of hospital stay for infants with bronchiolitis. J Investig Med. 2018;66:986–91. 29588331 10.1136/jim-2018-000708 PMC7269552

[4] Paes B, Fauroux B, Figueras-Aloy J, et al. Defining the risk and associated morbidity and mortality of severe respiratory syncytial virus infection among infants with Chronic Lung disease. Infect Dis Ther. 2016;5:453–71. 27864751 10.1007/s40121-016-0137-7 PMC5125140

[5] Checchia PA, Paes B, Bont L, et al. Defining the risk and associated morbidity and mortality of severe respiratory syncytial virus infection among infants with congenital heart disease. Infect Dis Ther. 2017;6:37–56. 28070870 10.1007/s40121-016-0142-x PMC5336417

[6] Figueras-Aloy J, Manzoni P, Paes B, et al. Defining the risk and associated morbidity and mortality of severe respiratory syncytial virus infection among preterm infants without Chronic Lung disease or congenital heart disease. Infect Dis Ther. 2016;5:417–52. 27628014 10.1007/s40121-016-0130-1 PMC5125133

[7] IMpact Study Group. Palivizumab, a humanized respiratory syncytial virus monoclonal antibody, reduces hospitalization from respiratory syncytial virus infection in high-risk infants. Pediatrics. 1998;102:531–37. 9724660

[8] Feltes TF, Cabalka AK, Meissner HC, et al. Palivizumab prophylaxis reduces hospitalization due to respiratory syncytial virus in young children with hemodynamically significant congenital heart disease. J Pediatr. 2003;143:532–40. 14571236 10.1067/s0022-3476(03)00454-2

[9] Mitchell I, Li A, Bjornson CL, Lanctot KL, Paes BA. CARESS investigators. Respiratory syncytial virus immunoprophylaxis with palivizumab: 12-year observational study of usage and outcomes in Canada. Am J Perinatol. 2022;39:1668–77. 33657636 10.1055/s-0041-1725146 PMC9643049

[10] Piñeros JG, De la Hoz-Valle J, Galvis C, et al. Effectiveness of palivizumab immunoprophylaxis in infants with respiratory syncytial virus disease in Colombia. J Infect Dev Ctries. 2021;15:1708–13. 34898500 10.3855/jidc.12561

[11] Rueda JD, Rosselli D, Ruiz-Pelaez JG. Cost-effectiveness of respiratory syncytial virus infection (RSV) prophylaxis with palivizumab in preterm infants in Colombia. Coyuntura Económica. 2013;43:137–51.

[12] Ministerio de Salud y Protección Social. Actualización de La Recomendación Sobre El Uso de Palivizumab. Available at: https://gpc.minsalud.gov.co/gpc_sites/Repositorio/Conv_500/GPC_rnp/gpc_rnp_completa.aspx. Accessed October 29, 2024.

[13] Ordóñez JE, Huertas VM. Cost-utility analysis of palivizumab for preventing respiratory syncytial virus in preterm neonates and infants in Colombia. BMC Infect Dis. 2024;24:418. 38641577 10.1186/s12879-024-09300-5 PMC11031882

[14] Blanken MO, Paes B, Anderson EJ, et al. Risk scoring tool to predict respiratory syncytial virus hospitalisation in premature infants. Pediatr Pulmonol. 2018;53:605–12. 29405612 10.1002/ppul.23960 PMC6099524

[15] Rodgers-Gray BS, Fullarton JR, Carbonell-Estrany X, Keary IP, Tarride JÉ, Paes BA. Impact of using the International risk scoring tool on the cost-utility of palivizumab for preventing severe respiratory syncytial virus infection in Canadian moderate-to-late preterm infants. J Med Econ. 2023;26:630–43. 37067826 10.1080/13696998.2023.2202600

[16] Keary IP, Ravasio R, Fullarton JR, et al. A new cost-utility analysis assessing risk factor-guided prophylaxis with palivizumab for the prevention of severe respiratory syncytial virus infection in Italian infants born at 29–35 weeks’ gestational age. PLoS One. 2023;18:e0289828. 37561741 10.1371/journal.pone.0289828 PMC10414677

[17] Gamba-Sanchez N, Rodriguez-Martinez CE, Sossa-Briceño MP. Epidemic activity of respiratory syncytial virus is related to temperature and rainfall in equatorial tropical countries. Epidemiol Infect. 2016;144:2057–63. 26888544 10.1017/S0950268816000273 PMC9150586

[18] Carbonell-Estrany X, Simões EAF, Fullarton JR, Ferdynus C, Gouyon J-B, Group ERRFS. Validation of a model to predict hospitalization due to RSV of infants born at 33–35 weeks’ gestation. J Perinat Med. 2010;38:411–17. 20297901 10.1515/jpm.2010.074

[19] Stensballe LG, Fullarton JR, Carbonell-Estrany X, Simões EAF. Population based external validation of a European predictive model for respiratory syncytial virus hospitalization of premature infants born 33 to 35 weeks of gestational age. Pediatr Infect Dis J. 2010;29:374–76. 20016397 10.1097/INF.0b013e3181c810da

[20] Simões EAF, Carbonell-Estrany X, Fullarton JR, et al. European risk factors’ model to predict hospitalization of premature infants born 33–35 weeks’ gestational age with respiratory syncytial virus: validation with Italian data. J Matern Fetal Neonatal Med. 2011;24:152–57. 20486882 10.3109/14767058.2010.482610

[21] Galvis C, Colmenares A, Cabrales L, et al. Impact of immunoprophylaxis with palivizumab on respiratory syncytial virus infection in preterm infants less than 35 weeks in Colombian hospitals. Pediatr Pulmonol. 2022;57:2420–27. 35791790 10.1002/ppul.26051

[22] Notario G, Vo P, Gooch K, et al. Respiratory syncytial virus-related hospitalization in premature infants without bronchopulmonary dysplasia: subgroup efficacy analysis of the IMpact-RSV trial by gestational age group. Pediatr Health Med Ther. 2014;5:43–48.

[23] Carbonell-Estrany X, Simões EAF, Dagan R, et al. Motavizumab for prophylaxis of respiratory syncytial virus in high-risk children: a noninferiority trial. Pediatrics. 2010;125:e35–51. 20008423 10.1542/peds.2008-1036

[24] Rodriguez-Martinez CE, Sossa-Briceño MP, Castro-Rodriguez JA. Direct medical costs of RSV-related bronchiolitis hospitalizations in a middle-income tropical country. Allergol Immunopathol (madr). 2020;48:56–61. 31235183 10.1016/j.aller.2019.04.004

[25] Weiner LB, Masaquel AS, Polak MJ, Mahadevia PJ. Cost-effectiveness analysis of palivizumab among pre-term infant populations covered by Medicaid in the United States. J Med Econ. 2012;15:997–1018. 22435648 10.3111/13696998.2012.672942

[26] Leidy NK, Margolis MK, Marcin JP, et al. The impact of severe respiratory syncytial virus on the child, caregiver, and family during hospitalization and recovery. Pediatrics. 2005;115:1536–46. 15930214 10.1542/peds.2004-1149

[27] Greenough A, Alexander J, Burgess S, et al. Health care utilisation of prematurely born, preschool children related to hospitalisation for RSV infection. Arch Dis Child. 2004;89:673–78. 15210503 10.1136/adc.2003.036129 PMC1720002

[28] Chiou CF, Weaver MR, Bell MA, Lee TA, Krieger JW. Development of the multiattribute pediatric asthma health outcome measure (PAHOM). Int J Qual Health Care. 2005;17:23–30. 15668307 10.1093/intqhc/mzh086

[29] Villamil JPS, Polack FP, Buendía JA. Disability-adjusted life years for respiratory syncytial virus in children under 2 years. BMC Public Health. 2020;20:1679. 33167966 10.1186/s12889-020-09796-x PMC7654061

[30] Carbonell-Estrany X, Pérez-Yarza EG, et al. Long-term burden and respiratory effects of respiratory syncytial virus hospitalization in preterm infants-the SPRING study. PLoS One. 2015;10:e0125422. 25955487 10.1371/journal.pone.0125422 PMC4425575

[31] Sigurs N, Bjarnason R, Sigurbergsson F, Kjellman B. Respiratory syncytial virus bronchiolitis in infancy is an important risk factor for asthma and allergy at age 7. Am J Respir Crit Care Med. 2000;161:1501–07. 10806145 10.1164/ajrccm.161.5.9906076

[32] Sigurs N, Gustafsson PM, Bjarnason R, et al. Severe respiratory syncytial virus bronchiolitis in infancy and asthma and allergy at age 13. Am J Respir Crit Care Med. 2005;171:137–41. 15516534 10.1164/rccm.200406-730OC

[33] Sigurs N, Aljassim F, Kjellman B, et al. Asthma and allergy patterns over 18 years after severe RSV bronchiolitis in the first year of life. Thorax. 2010;65:1045–52. 20581410 10.1136/thx.2009.121582

[34] Simoes E, Groothuis JR, Carbonell-Estrany X, et al. Palivizumab prophylaxis, respiratory syncytial virus, and subsequent recurrent wheezing. J Pediatr. 2007;151:34–42. 17586188 10.1016/j.jpeds.2007.02.032

[35] Blanken MO, Rovers MM, Molenaar JM, et al. Respiratory syncytial virus and recurrent wheeze in healthy preterm infants. N Engl J Med. 2013;368:1791–99. 23656644 10.1056/NEJMoa1211917

[36] Yoshihara S, Kusuda S, Mochizuki H, et al. Effect of palivizumab prophylaxis on subsequent recurrent wheezing in preterm infants. Pediatrics. 2013;132:811–18. 24127479 10.1542/peds.2013-0982

[37] Colombia Prices Paid Data. 2022 Available at: https://web.sispro.gov.co/WebPublico/Consultas/ConsultarCNPMCadenaComercializacionCircu2yPA_028_2_2.aspx. Accessed October 29, 2024.

[38] Economic Research Institute. Registered Nurse Salary in Colombia. 2022. Available: https://www.erieri.com/salary/job/registered-nurse/colombia#:%7E:text=The%20average%20pay%20for%20a,education%20for%20a%20Registered%20Nurse. Accessed October 29, 2024.

[39] Buendía JA, Patiño DG, Giraldo Ramírez JE. Cost utility of intermittent inhaled corticosteroids in preschoolers with viral-triggered wheeze. Pediatr Allergy Immunol Pulmonol. 2022;35:36–42. 35320007 10.1089/ped.2021.0143

[40] Flórez-Tanus Á, Parra D, Zakzuk J, Caraballo L, Alvis-Guzmán N. Health care costs and resource utilization for different asthma severity stages in Colombia: a claims data analysis. World Allergy Organ J. 2018;11:26. 30459927 10.1186/s40413-018-0205-4 PMC6231276

[41] Narayan O, Bentley A, Mowbray K, et al. Updated cost-effectiveness analysis of palivizumab (synagis) for the prophylaxis of respiratory syncytial virus in infant populations in the UK. J Med Econ. 2020;23:1640–52. 33107769 10.1080/13696998.2020.1836923

[42] Dirección de Censos y Demografía (DANE). Weeks of gestational age and weight 2021. Available at: https://www.dane.gov.co/index.php/acerca-del-dane/informacion-institucional/organigrama/direccion-de-censos-y-demografia. Accessed October 29, 2024.

[43] Statistics Canada. Birth statistics for the years 2016-2020. Canadian vital statistics, birth database. Ottawa (Canada): Statistics Canada; 2023.

[44] Instituto de Evaluación Tecnológica en Salud. Manual para la elaboración de evaluaciones económicas en salud. 2014. Available at: https://www.iets.org.co/Archivos/64/Manual_evaluacion_economica.pdf. Accessed October 29, 2024.

[45] The World Bank. GDP per capita. Available at: https://data.worldbank.org/indicator/NY.GDP.PCAP.CN?locations=CO. Accessed 14 September, 2023.

[46] Berger BA, Cossio A, Saravia NG, et al. Cost-effectiveness of meglumine antimoniate versus miltefosine caregiver DOT for the treatment of pediatric cutaneous leishmaniasis. PLoS Negl Trop Dis. 2017;11:e0005459. 28384261 10.1371/journal.pntd.0005459 PMC5404883

[47] Sheridan-Pereira M, Murphy J, Sloan J, et al. Respiratory syncytial virus preterm (32-36 completed weeks’ gestation) risk estimation measure for RSV hospitalization in Ireland: a prospective study. Pediatr Infect Dis J. 2016;35:19–24. 26379160 10.1097/INF.0000000000000918

[48] Carbonell-Estrany X, Rodgers-Gray BS, Paes B. Challenges in the prevention or treatment of RSV with emerging new agents in children from low- and middle-income countries. Expert Rev Anti Infect Ther. 2021;19:419–41. 32972198 10.1080/14787210.2021.1828866

[49] Thwaites R, Buchan S, Fullarton J, et al. Clinical burden of severe respiratory syncytial virus infection during the first 2 years of life in children born between 2000 and 2011 in Scotland. Eur J Pediatr. 2020;179:791–99. 31912234 10.1007/s00431-019-03564-9 PMC7160099

[50] Paes B, Lanari M, Rodgers-Gray B, Fullarton J, Carbonell-Estrany X. Opinion: the optimal use of risk factors to guide palivizumab prophylaxis against severe respiratory syncytial virus infection in moderate-to-late preterm infants. Front Pediatr. 2024;12:1343960. 38283631 10.3389/fped.2024.1343960 PMC10811053

[51] Carbonell-Estrany X, Madihlabab T, Rodgers-Gray B, Vigna N, Fullarton J. Provision of additional risk factors to facilitate country utilization of the risk scoring tool (RST) for predicting respiratory syncytial virus hospitalisation (RSVH) in moderate-to-late preterm infants. Presented at: 9th International Conference on Clinical Neonatology (ICCN). Turin: 2020 September 3–5.

[52] World Health Organization. Breastfeeding. 2024. Available at: https://www.who.int/health-topics/breastfeeding#tab=tab_2. Accessed October 29, 2024.

[53] Coutts J, Fullarton J, Morris C, et al. Association between respiratory syncytial virus hospitalization in infancy and childhood asthma. Pediatr Pulmonol. 2020;55:1104–10. 32040885 10.1002/ppul.24676 PMC7187471

[54] Anderson EJ, DeVincenzo JP, Simões EAF, et al. SENTINEL1: two-season study of respiratory syncytial virus hospitalizations among U.S. Infants born at 29 to 35 weeks’ gestational age not receiving immunoprophylaxis. Am J Perinatol. 2020;37:421–29. 30991438 10.1055/s-0039-1681014

[55] Arruda E, Jones MH, Escremim de Paula F, et al. The burden of single virus and viral coinfections on severe lower respiratory tract infections among preterm infants: a prospective birth cohort study in Brazil. Pediatr Infect Dis. 2014;J 33:997–1003. 10.1097/INF.0000000000000349 25361184

[56] Ochoa TJ, Bautista R, Davila C, et al. Respiratory syncytial virus-associated hospitalizations in pre-mature infants in Lima, Peru. Am J Trop Med Hyg. 2014;91:1029–34. 25294617 10.4269/ajtmh.13-0648 PMC4228870

[57] Hodgson D, Atkins KE, Baguelin M, et al. Estimates for quality of life loss due to respiratory syncytial virus. Influenza Other Respir Viruses. 2020;14:19–27. 31625688 10.1111/irv.12686 PMC6928035

[58] Díez-Gandıa E, Gómez-Alvarez C, López-Lacort M, et al. The impact of childhood RSV infection on children’s and parents’ quality of life: a prospective multicenter study in Spain. BMC Infect Dis. 2021;21:924. 34488668 10.1186/s12879-021-06629-z PMC8422742

[59] Sheng-Kai Ma K, Tsai SY, El Saleeby CM, Kotton CN, Mansbach JM. Nirsevimab decreased the subsequent risk of respiratory syncytial virus infection and wheezing in the 2023–2024 RSV season. Pediatr Res. 2025;98:388–90. 39789206 10.1038/s41390-024-03782-4

[60] Central Bank of Colombia. Available: https://www.banrep.gov.co/es. Accessed October 29, 2024.

[61] Pichon-Riviere A, Drummond M, Palacios A, Garcia-Marti S, Augustovski F. Determining the efficiency path to universal health coverage: cost-effectiveness thresholds for 174 countries based on growth in life expectancy and health expenditures. Lancet Glob Health. 2023;11:e833–42. 37202020 10.1016/S2214-109X(23)00162-6

[62] Espinosa O, Rodríguez-Lesmes P, Orozco L, et al. Estimating cost-effectiveness thresholds under a managed healthcare system: experiences from Colombia. Health Policy Plan. 2022;37:359–68. 34875689 10.1093/heapol/czab146

[63] World Health Organization. Threshold values for intervention cost-effectiveness by region. In: Choosing interventions that are cost effective. (WHO-CHOICE). World Health Organization; 2010.

