# Supplementary material for: Clinical and economic evaluation of risk factor guided respiratory syncytial virus prophylaxis in Colombian preterm infants
Source: Cost Eff Resour Alloc. 2026 Jan 27;24:35. doi: 10.1186/s12962-025-00710-z (PMC12918000; doi:10.1186/s12962-025-00710-z)
Supplement: Supplementary file 1 — Supplementary material 1 [file 12962_2025_710_MOESM1_ESM.docx]

Clinical and economic evaluation of risk factor guided respiratory syncytial virus prophylaxis in Colombian preterm infants

Carlos E. Rodriguez-Martinez, Jaime Ordonez, Xavier Carbonell-Estrany, John Fullarton, Ian Keary, Barry Rodgers-Gray, Ivonne D’Apremont, Daniel E. Noyola, Paulo Andre Ribeiro, Renato T. Stein, Nestor Vain, Jean-Eric Tarride, Bosco Paes

*The Journal of Infection in Developing Countries*

Corresponding author:

Barry S Rodgers-Gray, Violicom Medical Limited, 1 Andromeda House Calleva Park, Aldermaston, United Kingdom. RG7 8AP.

[barry@violicom.co.uk](mailto:barry@violicom.co.uk)

Supplementary tables and figures

**Table S1** Source data and predictive accuracy of the International Risk Scoring Tool^[[1]](#endnote-2)^

| **Source data** | **AUROC**  **(Sensitivity; Specificity)** |
| --- | --- |
| *- Development* (N=13475): PICNIC (Canada); FLIP-2 (Spain); RISK (Netherlands); REPORT (USA); IBC (Italy); PONI (Europe, Central America, Asia & Middle East)  *- Validation* (N=1078): PREMI (Ireland) | 0.773  (68.9%; 73.0%) |

AUROC: area under the receiver operating characteristic curve, where 1 equals perfect predictive accuracy; FLIP: Risk Factors Linked to RSV Infection Requiring Hospitalization in Premature Infants study; IBC: Italian Birth Cohort; PICNIC: Pediatric Investigators Collaborative Network on Infections in Canada study; PONI: Predictors Associated with RSV Hospitalization in Non-prophylaxed, Premature Infants study; PREMI: Preterm Risk Estimation Measure for RSVH in Ireland study; REPORT: Respiratory Events Among Preterm Infants Outcomes and Risk Tracking study; RISK: RISK study; RSVH: RSV-related hospitalization; RST: risk scoring tool; sensitivity: proportion of RSVHs correctly identified; specificity: proportion of non-RSVHs correctly identified

**References**

**Table S2** Gestational age as a substitute for chronological age in the International Risk Scoring Tool (IRST)

| **Weeks’ gestational age** | **Hospitalized (n=484)** | **Non-hospitalized (n=12,991)** | **Odds Ratio (95% CI)** | **P-value*** |
| --- | --- | --- | --- | --- |
| Mean, SD | 34.2 (0.9) | 34.2 (0.9) | - | 0.922 (NS) |
| 32^0^–32^6^ | 40 (8.3%) | 867 (6.7%) | 1.3 (0.9–1.8) | 0.171 (NS) |
| 33^0^–33^6^ | 116 (24.0%) | 3,168 (24.4%) | 1.0 (0.8–1.2) | 0.833 (NS) |
| 34^0^–34^6^ | 187 (38.6%) | 4,949 (38.1%) | 1.0 (0.8–1.2) | 0.810 (NS) |
| 35^0^–35^6^ | 141 (29.1%) | 4,007 (30.8%) | 0.9 (0.8–1.1) | 0.423 (NS) |

*Two-tailed T-test; Fisher exact test (2 tailed). CI: confidence interval; NS: not significant; SD: standard deviation

Analysis of the pooled dataset underpinning the IRST^[[2]](#endnote-3)^ revealed no significant differences across weeks’ gestational age (wGA) for respiratory syncytial virus hospitalization risk, though an expected trend towards increased risk at lower wGA

**References**

**Table S3** Risk groups in the International Risk Scoring Tool^[[3]](#endnote-4)^

| **Risk Score** | **Proportion of infants in risk group** | **Proportion of all RSVHs** | **RSVH rate (%)** |
| --- | --- | --- | --- |
| **Low: ≤19**  **Moderate: 20-45**  **High: 50-56** | 51.3%  25.1%  23.67% | 14.8%  23.2%  62.0% | 1.0%  3.3%  9.5% |

RSVH: respiratory syncytial virus hospitalization

**References**

**Table S4** Rates of long-term respiratory morbidity

| **Year** | **Palivizumab** | | **No Palivizumab** | | **Source(s)** |
| --- | --- | --- | --- | --- | --- |
|  | **RSVH** | **No RSVH** | **RSVH** | **No RSVH** |  |
| **0–1** | 18.4% | 5.4% | 41.4% | 12.1% | Respiratory morbidity from SPRING study (Carbonell-Estrany *et al*. 2015^[[4]](#endnote-5)^), as modified by Sanchez Luna *et al*. 2017^[[5]](#endnote-6)^ to infer palivizumab efficacy using data from Blanken *et al* 2013^[[6]](#endnote-7)^, Simoes *et al*. 2007^[[7]](#endnote-8)^ & Yoshihara *et al*. 2013^[[8]](#endnote-9)^ |
| **1–2** | 18.4% | 5.4% | 41.4% | 12.1% |  |
| **2–3** | 11.1% | 5.8% | 29.3% | 15.4% |  |
| **3–4** | 6.1% | 4.2% | 18.6% | 12.6% |  |
| **4–5** | 4.4% | 2.7% | 15.0% | 9.3% |  |
| **5–6** | 3.3% | 2.5% | 12.4% | 9.7% |  |
| **6–7** | 2.9% | 2.3% | 12.4% | 9.7% | Sigurs *et al*. 2000^[[9]](#endnote-10)^ adjusted to fit SPRING^1^ and modified as above |
| **7–13** | 2.3% | 1.5% | 17.4% | 11.0% | Sigurs *et al*. 2005^[[10]](#endnote-11)^ adjusted to fit SPRING^1^ and modified as above |
| **13–18** | 1.8% | 1.2% | 22.4% | 14.7% | Sigurs *et al*. 2010^[[11]](#endnote-12)^ adjusted to fit SPRING^1^ and modified as above |

RSV: respiratory syncytial virus; RSVH: RSV-related hospitalization

For infants with RSVH, long-term respiratory morbidity rates up to 18 years are displayed in Table S4. For infants with medically-attended RSV infection (MARI), the corresponding rates were taken from those labelled ‘No RSVH’ in Table S4. Infants without an RSV infection or an RSV infection not requiring any medical management were assumed to have some background respiratory morbidity for up to 6 years, using the same rates as for MARI.

**References**

**Table S5** Probability distributions used in probabilistic sensitivity analysis

| **Parameter** | **Mean** | **SD** | **Min** | **Max** | **Distribution** | **Source** |
| --- | --- | --- | --- | --- | --- | --- |
| Annual cost of respiratory sequelae | 1,630,443.89 | 163,044.39 | 1,304,355.11 | 1,956,532.67 | Gamma | ^[[12]](#endnote-13)^,^[[13]](#endnote-14)^ |
| Average birthweight | 1,991.20 | 199.12 | 1,592.96 | 2,389.44 | Normal | ^[[14]](#endnote-15)^,^[[15]](#endnote-16)^ |
| Background utility score | 0.950 | 0.10 | 0.76 | 1.00 | Beta | ^[[16]](#endnote-17)^ |
| Clinician consultation cost | 32,450.23 | 3,245.02 | 25,960.19 | 38,940.28 | Gamma | ^[[17]](#endnote-18)^ |
| Cost per 100mg vial | 2,804,887.19 | 280,488.72 | 2,243,909.75 | 3,365,864.63 | Gamma | ^[[18]](#endnote-19)^ |
| Cost per 50mg vial | 1,416,724.00 | 141,672.40 | 1,133,379.20 | 1,700,068.80 | Gamma | 7 |
| Discount rate costs | 5.00% | 0.50% | 4.00% | 6.00% | Normal | ^[[19]](#endnote-20)^ |
| Discount rate utilities | 5.00% | 0.50% | 4.00% | 6.00% | Normal | 8 |
| Disutility whilst in hospital | 0.6 | 0.06 | 0.48 | 0.72 | Beta | ^[[20]](#endnote-21)^,^[[21]](#endnote-22)^ |
| ED visit cost | 69,663.41 | 6,966.34 | 55,730.73 | 83,596.09 | Gamma | 6 |
| Hospital length of stay 32-35wGA | 8.6 | 0.86 | 6.88 | 10.32 | Normal | ^[[22]](#endnote-23)^ |
| Hospitalization cost bundle, including ICU | 7,240,740.14 | 724,074.01 | 5,792,592.11 | 8,688,888.17 | Gamma | 6 |
| ICU rate PVZ | 7.80% | 0.78% | 6.24% | 9.36% | Beta | 6 |
| LT morbidity rates no PVZ after RSV (multiplier) | 1 | 0.1 | 0.8 | 1.2 | Beta | ^[[23]](#endnote-24)^,^[[24]](#endnote-25)^,^[[25]](#endnote-26)^,^[[26]](#endnote-27)^ |
| LT morbidity rates no PVZ no RSV (multiplier) | 1 | 0.1 | 0.8 | 1.2 | Beta | 12,13,1415 |
| LT morbidity rates PVZ after RSV (multiplier) | 1 | 0.1 | 0.8 | 1.2 | Beta | 12,13,1415,^[[27]](#endnote-28)^,^[[28]](#endnote-29)^,^[[29]](#endnote-30)^ |
| LT morbidity rates PVZ no RSV (multiplier) | 1 | 0.1 | 0.8 | 1.2 | Beta | 12,13,1415,16,1718 |
| MARI rates PVZ ED only | 2.95% | 0.30% | 2.36% | 3.54% | Beta | ^[[30]](#endnote-31)^ |
| Mean number of injections given | 4 | 0.4 | 3.2 | 4.8 | Normal | ^[[31]](#endnote-32)^ |
| Mortality no PVZ | 3.60% | 0.36% | 2.88% | 4.32% | Normal | ^[[32]](#endnote-33)^ |
| Mortality PVZ | 3.60% | 0.36% | 2.88% | 4.32% | Normal | 21 |
| Mortality rate | 3.60% | 0.36% | 2.88% | 4.32% | Normal | 21 |
| Non-prophylaxed first hospitalization rate | 18.60% | 1.86% | 14.88% | 22.32% | Beta | ^[[33]](#endnote-34)^ |
| Non-prophylaxed ICU rate | 7.80% | 0.78% | 6.24% | 9.36% | Beta | 6 |
| Non-prophylaxed second hospitalization rate | 12.42% | 1.24% | 9.93% | 14.90% | Beta | 22,^[[34]](#endnote-35)^,^[[35]](#endnote-36)^ |
| Palivizumab relative risk reduction | 0.82 | 0.08 | 0.66 | 0.9 | Normal | ^[[36]](#endnote-37)^ |
| RSV hospitalized utility score | 0.88 | 0.09 | 0.7 | 1 | Beta | 5 |
| Subsequent RSV rate PVZ | 12.42% | 1.24% | 9.93% | 14.90% | Beta |  |
| Utility with significant respiratory sequelae | 0.79 | 0.08 | 0.63 | 0.95 | Beta | ^[[37]](#endnote-38)^ |

All costs listed in Colombian peso

ED: emergency department; ICU: intensive care unit; LOS: length of stay; LT: long term; MARI: medically attended RSV infection; PVZ: palivizumab; RSV: respiratory syncytial virus; SD: standard deviation; wGA: weeks’ gestational age

**References**

**Table S6** Indirect costs for cost-utility model

| Parameter | Cost (COP) | Units | Reference source(s) |
| --- | --- | --- | --- |
| **Palivizumab administration**  - Transport  - Missed work | 0  0 | Assumes palivizumab given during routine “kangaroo care” program visit | N/A  N/A |
| **RSVH**  - Missed work  - Childcare^a^  - Transport^a^  - Other out of pocket expenses^a^ | 1,370071.50  71,453.00  116,515.07  474,267.36 | 1 *per* infant with RSVH^b^ | Berger *et al.* 2017^[[38]](#endnote-39)^  Berger *et al.* 2017^1^  Berger *et al.* 2017^1^  Berger *et al.* 2017^1^ |
| **MARI attendance**  - Transport  - Missed work | 13,548.26  39,287.66 | 1 *per* infant with MARI^b^ | Berger *et al.* 2017^1^  Berger *et al.* 2017^1^ |
| **Loss of earnings following death** | 1,550,360,000.00 | 1 *per* infant suffering mortality^b^ | Salary explorer^[[39]](#endnote-40)^ and The World Bank^[[40]](#endnote-41)^ |

^a^Costs uplifted to Colombia 2022 levels using the latest World Bank Data available at the time of writing. ^b^See Table 1 for rates. COP: Colombian peso; MARI: medically-attended RSV infection without RSVH; RSVH: respiratory syncytial virus hospitalization.

**References**

**Table S7** Colombian risk factor data

| **Variable** | **n** | **Cases**  **(n=81)** | **Controls**  **(n=49)** | **p-value*** |
| --- | --- | --- | --- | --- |
| **32 weeks gestational age** | 17 | 7 | 10 | 0.292 |
| **33 weeks gestational age** | 24 | 16 | 8 |  |
| **34 weeks gestational age** | 35 | 22 | 13 |  |
| **35 weeks gestational age** | 54 | 36 | 18 |  |
|  | | | | |
| **Smokers in house other than mother – Yes** | 8 | 6 | 2 | 0.709 |
| **Smokers in house other than mother – No** | 115 | 72 | 43 |  |
|  | | | | |
| **Presence of non-twin siblings – Yes** | 40 | 35 | 5 | **0.001** |
| **Presence of non-twin siblings – No** | 67 | 38 | 29 |  |
|  | | | | |
| **Attended daycare – Yes** | 12 | 9 | 3 | 0.531 |
| **Attended daycare – No** | 103 | 64 | 39 |  |
|  | | | | |
| **Maternal education to primary level or less – Yes** | 26 | 14 | 12 | 1.000 |
| **Maternal education to primary level or less – No** | 58 | 32 | 26 |  |
|  | | | | |
| **Breast fed – Yes** | 117 | 72 | 45 | 0.256 |
| **Breast fed – No** | 8 | 7 | 1 |  |
|  | | | | |
| **Breastfeeding mixed with formula – Yes** | 78 | 45 | 33 | 0.160 |
| **Breastfeeding mixed with formula – No** | 39 | 28 | 11 |  |
|  | | | | |
| **Maternal smoking while pregnant – Yes** | 5 | 4 | 1 | 0.651 |
| **Maternal smoking while pregnant – No** | 118 | 74 | 44 |  |

^*^Calculated using the Fisher exact test (2 tailed)

**Figure S1** International Risk Scoring Tool – risk factors and scores^[[41]](#endnote-42)^


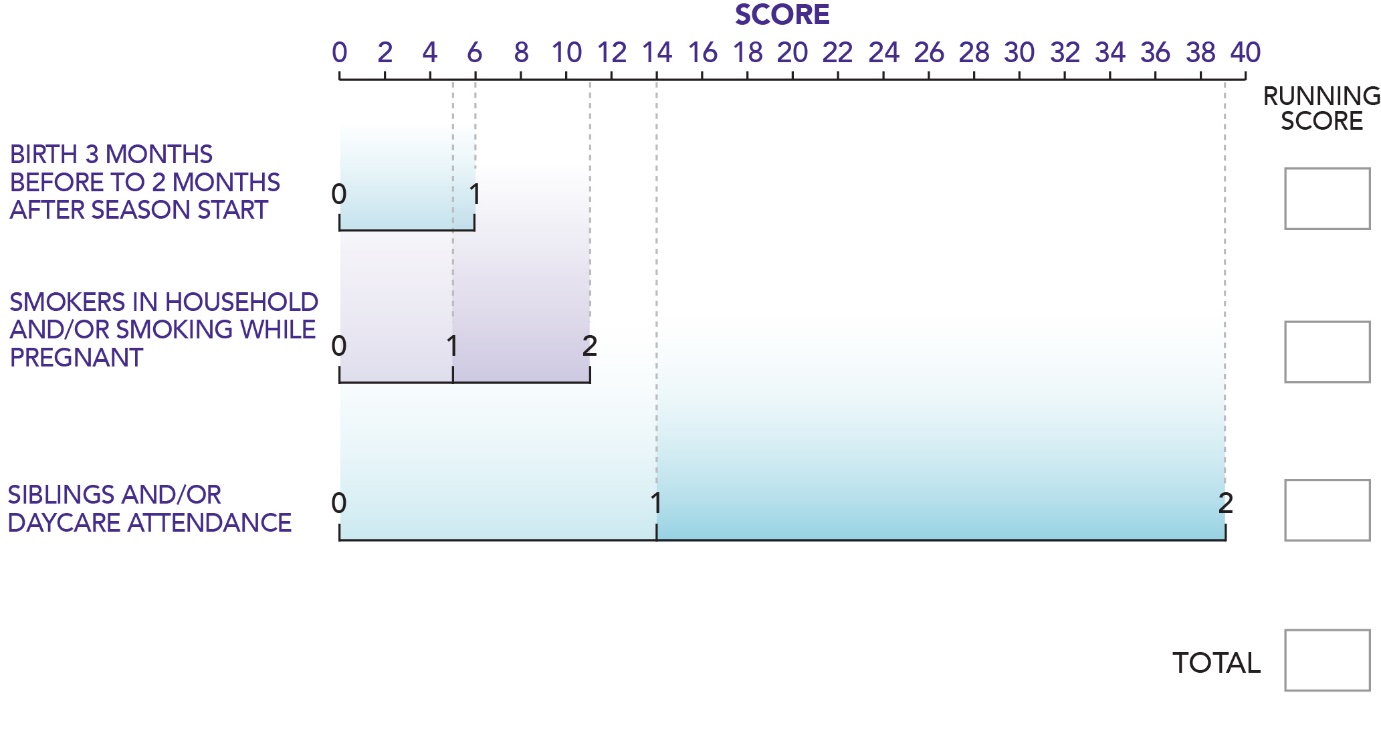


**References**

**Figure S2** IRST generated from Colombian data with ‘birth 3 months before to 2 months after the RSV season start date’ replaced by gestational age

**AUROC: 0.751**

Included risk factors: Gestational age; smokers and/or maternal smoking; siblings and/or daycare attendance

AUROC: Area under the receiver operating characteristic curve, with 1 representing perfect predictive accuracy; IRST: International Risk Scoring Tool; RSV: respiratory syncytial virus

**Figure S3** IRST generated from Colombian data with lack of exclusive breast feeding included

**AUROC: 0.791**

Included risk factors: Gestational age; smokers and/or maternal smoking; siblings and/or daycare; lack of breastfeeding (defined as exclusive and given or planned from birth to 3 months of age)

AUROC: Area under the receiver operating characteristic curve, with 1 representing perfect predictive accuracy; IRST: International Risk Scoring Tool

**Figure S4** IRST generated from Colombian data with mixed breastfeeding included

**AUROC: 0.803**

Included risk factors: Gestational age; smokers and/or maternal smoking; siblings and/or daycare; mixed breastfeeding (breastfeeding plus formula)

AUROC: Area under the receiver operating characteristic curve, with 1 representing perfect predictive accuracy; IRST: International Risk Scoring Tool

**Figure S5** Incremental cost-effectiveness plane for palivizumab prophylaxis (*vs* no prophylaxis) in moderate- and high-risk 32–35 wGA infants identified by the Colombian-specific IRST

Results are based on the probabilistic sensitivity analysis after 10,000 Monte Carlo simulations. The solid line represents an acceptability threshold of $84,581,202/QALY. COP: Colombian peso; IRST: international risk scoring tool; PSA: probabilistic sensitivity analysis; QALY: quality-adjusted life year; wGA: weeks’ gestational age; WTP: willingness-to-pay threshold

**Figure S6** One-way deterministic sensitivity analysis (±20%) for palivizumab *versus* no prophylaxis in the base case (15 most sensitive variables)

Hospitalization

hospitalization

Costs expressed in Colombian Pesos. ED: emergency department; ICU: intensive care unit; LT: long-term; MARI: medically-attended RSV infection without RSV hospitalization; mg: milligram; PVZ: palivizumab; RSV: respiratory syncytial virus; wGA: weeks‘ gestational age

1. Blanken MO, Paes B, Anderson EJ, Lanari M, Sheridan-Pereira M, Buchan S, et al. Risk scoring tool to predict respiratory syncytial virus hospitalisation in premature infants. Pediatr Pulmonol. 2018;53(5):605–612. [↑](#endnote-ref-2)
2. Blanken MO, Paes B, Anderson EJ, Lanari M, Sheridan-Pereira M, Buchan S, et al. Risk scoring tool to predict respiratory syncytial virus hospitalisation in premature infants. Pediatr Pulmonol. 2018;53(5):605–612. [↑](#endnote-ref-3)
3. Blanken MO, Paes B, Anderson EJ, Lanari M, Sheridan-Pereira M, Buchan S, et al. Risk scoring tool to predict respiratory syncytial virus hospitalisation in premature infants. Pediatr Pulmonol. 2018;53(5):605–612. [↑](#endnote-ref-4)
4. Carbonell-Estrany X, Pérez-Yarza EG, Sanchez García L, et al. Long-Term Burden and Respiratory Effects of Respiratory Syncytial Virus Hospitalization in Preterm Infants-The SPRING Study. *PLoS One* 2015;10(5):e0125422. [↑](#endnote-ref-5)
5. Sanchez-Luna M, Burgos-Pol R, Oyagüez I, et al. Cost-utility analysis of Palivizumab for Respiratory Syncytial Virus infection prophylaxis in preterm infants: update based on the clinical evidence in Spain. *BMC Infect Dis*. 2017;17(1):687. [↑](#endnote-ref-6)
6. Blanken MO, Rovers MM, Molenaar JM, et al. Respiratory syncytial virus and recurrent wheeze in healthy preterm infants. N Engl J Med 2013;368(19):1791–1799. [↑](#endnote-ref-7)
7. Simoes E, Groothuis JR, Carbonell-Estrany X, et al. Palivizumab prophylaxis, respiratory syncytial virus, and subsequent recurrent wheezing. *J Pediatr*. 2007;151(1):34–42. [↑](#endnote-ref-8)
8. Yoshihara S, Kusuda S, Mochizuki H, et al. Effect of palivizumab prophylaxis on subsequent recurrent wheezing in preterm infants. Pediatrics 2013;132(5):811–818. [↑](#endnote-ref-9)
9. Sigurs N, Bjarnason R, Sigurbergsson F, Kjellman B. Respiratory syncytial virus bronchiolitis in infancy is an important risk factor for asthma and allergy at age 7. *Am J Respir Crit Care Med*. 2000*;*161(5):1501-1507. [↑](#endnote-ref-10)
10. Sigurs N, Gustafsson PM, Bjarnason R, et al. Severe respiratory syncytial virus bronchiolitis in infancy and asthma and allergy at age 13. Am J Respir Crit Care Med 2005;171(2):137-141. [↑](#endnote-ref-11)
11. Sigurs N, Aljassim F, Kjellman B, et al. Asthma and allergy patterns over 18 years after severe RSV bronchiolitis in the first year of life. *Thorax*. 2010;65(12):1045–1052. [↑](#endnote-ref-12)
12. Flórez-Tanus Á, Parra D, Zakzuk J, Caraballo L, Alvis-Guzmán N. Health care costs and resource utilization for different asthma severity stages in Colombia: a claims data analysis. World Allergy Organ J. 2018;11(1):26. [↑](#endnote-ref-13)
13. Buendía JA, Patiño DG, Giraldo Ramírez JE. Cost Utility of Intermittent Inhaled Corticosteroids in Preschoolers with Viral-Triggered Wheeze. Pediatr Allergy Immunol Pulmonol 2022;35:36–42. [↑](#endnote-ref-14)
14. Dirección de Censos y Demografía (DANE). Weeks of Gestational Age and Weight, 2021. <https://www.dane.gov.co/index.php/acerca-del-dane/informacion-institucional/organigrama/direccion-de-censos-y-demografia>. Accessed March 2024. [↑](#endnote-ref-15)
15. Statistics Canada. Canadian Vital statistics, birth database. Birth statistics for the years 2016–2020. Ottawa (Canada): Statistics Canada. [↑](#endnote-ref-16)
16. Greenough A, Alexander J, Burgess S, Bytham J, Chetcuti PA, Hagan J, et al. Health care utilisation of prematurely born, preschool children related to hospitalisation for RSV infection. Arch Dis Child. 2004;89(7):673–78. [↑](#endnote-ref-17)
17. Rodriguez-Martinez CE, Sossa-Briceño MP, Castro-Rodriguez JA. Direct medical costs of RSV-related bronchiolitis hospitalizations in a middle-income tropical country. Allergol Immunopathol (Madr). 2020;48(1):56-61. [↑](#endnote-ref-18)
18. Colombia Prices Paid Data. <https://web.sispro.gov.co/WebPublico/Consultas/ConsultarCNPMCadenaComercializacionCircu2yPA_028_2_2.aspx>. Accessed March 2024. [↑](#endnote-ref-19)
19. Instituto de Evaluación Tecnológica en Salud. Manual para la elaboración de evaluaciones económicas en salud. <https://www.iets.org.co/Archivos/64/Manual_evaluacion_economica.pdf>. Accessed September 2023. [↑](#endnote-ref-20)
20. Weiner LB, Masaquel AS, Polak MJ, Mahadevia PJ. Cost-effectiveness analysis of palivizumab among pre-term infant populations covered by Medicaid in the United States. J Med Econ. 2012;15(5):997–1018. [↑](#endnote-ref-21)
21. Leidy NK, Margolis MK, Marcin JP, Flynn JA, Frankel LR, Johnson S, et al. The impact of severe respiratory syncytial virus on the child, caregiver, and family during hospitalization and recovery. Pediatrics. 2005;115(6):1536–46. [↑](#endnote-ref-22)
22. Piñeros JG, Baquero H, Bastidas J, et al. Respiratory syncytial virus infection as a cause of hospitalization in population under 1 year in Colombia. J Pediatr (Rio J). 2013;89(6):544–548. [↑](#endnote-ref-23)
23. Carbonell-Estrany X, Pérez-Yarza EG, Sanchez García L, Guzmán Cabañas JM, Bòria EV, Atienza BB; IRIS (Infección Respiratoria Infantil por Virus Respiratorio Sincitial) Study Group. Long-Term Burden and Respiratory Effects of Respiratory Syncytial Virus Hospitalization in Preterm Infants-The SPRING Study. PLoS One. 2015;10(5):e0125422. [↑](#endnote-ref-24)
24. Sigurs N, Bjarnason R, Sigurbergsson F, Kjellman B. Respiratory syncytial virus bronchiolitis in infancy is an important risk factor for asthma and allergy at age 7. Am J Respir Crit Care Med. 2000;161(5):1501–07. [↑](#endnote-ref-25)
25. Sigurs N, Gustafsson PM, Bjarnason R, Lundberg F, Schmidt S, Sigurbergsson F, et al. Severe respiratory syncytial virus bronchiolitis in infancy and asthma and allergy at age 13. Am J Respir Crit Care Med. 2005;171(2):137–41. [↑](#endnote-ref-26)
26. Sigurs N, Aljassim F, Kjellman B, Robinson PD, Sigurbergsson F, Bjarnason R, et al. Asthma and allergy patterns over 18 years after severe RSV bronchiolitis in the first year of life. Thorax. 2010;65(12):1045–52. [↑](#endnote-ref-27)
27. Simoes E, Groothuis JR, Carbonell-Estrany X, Rieger CH, Mitchell I, Fredrick LM, et al. Palivizumab prophylaxis, respiratory syncytial virus, and subsequent recurrent wheezing. J Pediatr. 2007;151(1):34–42. [↑](#endnote-ref-28)
28. Blanken MO, Rovers MM, Molenaar JM, Winkler-Seinstra PL, Meijer A, Kimpen JL, Dutch RSV Neonatal Network. Respiratory syncytial virus and recurrent wheeze in healthy preterm infants. N Engl J Med. 2013;368(19):1791–1799. [↑](#endnote-ref-29)
29. Yoshihara S, Kusuda S, Mochizuki H, Okada K, Nishima S, Simões EA, C-CREW Investigators. Effect of palivizumab prophylaxis on subsequent recurrent wheezing in preterm infants. Pediatrics. 2013;132(5):811–818. [↑](#endnote-ref-30)
30. Carbonell-Estrany X, Simões EAF, Dagan R, Hall CB, Harris B, Hultquist M, et al. Motavizumab for prophylaxis of respiratory syncytial virus in high-risk children: a noninferiority trial. Pediatrics. 2010;125(1):e35–51. [↑](#endnote-ref-31)
31. Rodriguez-Martinez CE. Clinical experience. Personal Communication. March 2024. [↑](#endnote-ref-32)
32. Villamil JPS, Polack FP, Buendía JA. Disability-adjusted life years for respiratory syncytial virus in children under 2 years. BMC Public Health. 2020;20(1):1679. [↑](#endnote-ref-33)
33. Blanken MO, Paes B, Anderson EJ, Lanari M, Sheridan-Pereira M, Buchan S, et al. Risk scoring tool to predict respiratory syncytial virus hospitalisation in premature infants. Pediatr Pulmonol. 2018;53(5):605–612. [↑](#endnote-ref-34)
34. Figueras-Aloy J, Carbonell-Estrany X, Quero-Jiménez J, Fernández-Colomer B, Guzmán-Cabañas J, Echaniz-Urcelay I, IRIS Study Group. FLIP-2 Study: risk factors linked to respiratory syncytial virus infection requiring hospitalization in premature infants born in Spain at a gestational age of 32 to 35 weeks. Pediatr Infect Dis J. 2008;27:788–93. [↑](#endnote-ref-35)
35. Lanari M, Anderson EJ, Sheridan-Pereira M, Carbonell-Estrany X, Paes B, Rodgers-Gray BS, et al. Burden of respiratory syncytial virus hospitalisation among infants born at 32-35 weeks' gestational age in the Northern Hemisphere: pooled analysis of seven studies. Epidemiology and Infection 2020; 148:e170. [↑](#endnote-ref-36)
36. Notario G, Vo P, Gooch K, Deaton R, Wu X, Harris B, et al. Respiratory syncytial virus-related hospitalization in premature infants without bronchopulmonary dysplasia: subgroup efficacy analysis of the IMpact-RSV trial by gestational age group. Pediatric Health Med Ther. 2014;5:43–48. [↑](#endnote-ref-37)
37. Chiou CF, Weaver MR, Bell MA, Lee TA, Krieger JW. Development of the multiattribute pediatric asthma health outcome measure (PAHOM). Int J Qual Health Care. 2005;17(1):23–30. [↑](#endnote-ref-38)
38. Berger BA, Cossio A, Saravia NG, Del Mar Castro M, Prada S, Bartlett AH, Pho MT. Cost-effectiveness of meglumine antimoniate versus miltefosine caregiver DOT for the treatment of pediatric cutaneous leishmaniasis. PLoS Negl Trop Dis 2017;11:e0005459. [↑](#endnote-ref-39)
39. Salary explorer. Average Salary in Colombia. <https://www.salaryexplorer.com/average-salary-wage-comparison-colombia-c47>. Accessed September 2023. [↑](#endnote-ref-40)
40. The World Bank. Employment to population ratio – Colombia. <https://data.worldbank.org/indicator/SL.EMP.TOTL.SP.ZS?locations=CO>. Accessed September 2023. [↑](#endnote-ref-41)
41. Blanken MO, Paes B, Anderson EJ, Lanari M, Sheridan-Pereira M, Buchan S, et al. Risk scoring tool to predict respiratory syncytial virus hospitalisation in premature infants. Pediatr Pulmonol. 2018;53(5):605–612. [↑](#endnote-ref-42)
